# Surgical Treatment of Persistent Glue Extension Into Common Femoral Vein Following Endovenous Glue Ablation

**DOI:** 10.7759/cureus.56364

**Published:** 2024-03-18

**Authors:** Musaad AlHamzah

**Affiliations:** 1 Department of Surgery, King Saud University, Riyadh, SAU

**Keywords:** open surgical repair, high ligation, complication, glue emobolisation complication, vein ablation

## Abstract

Endovenous glue-induced thrombosis (EGIT) is a known complication of endovenous ablation therapy using cyanoacrylate closure glue to treat saphenous insufficiency, where glue extends into the common femoral vein (CFV), causing partial or complete occlusion. We report a case of class III EGIT (over 50% occlusion of CFV) in a young, healthy female who could not tolerate medical therapy. EGIT was treated with high ligation of the great saphenous vein and CFV patch repair. This is a safe option for patients who cannot tolerate anticoagulation therapy due to complications, physical or psychological limitations, or both.

## Introduction

Cyanoacrylate closure (CAC) glue is a non-thermal, non-tumescent therapy that is used for the treatment of saphenous insufficiency and has been used for over a decade [1]. Using CAC has gained popularity among physicians and patients due to its high closure rates, as well as the elimination of tumescent use and postoperative compression therapy [2,3]. However, glue extension into the deep venous system (also known as endovenous glue-induced thrombosis, EGIT) has been reported in 5.8-21% of cases [3]. This report presents a case of the extension of glue into the deep venous system and surgical therapy as a treatment option for this complication.

## Case presentation

The patient was an otherwise healthy 32-year-old woman who underwent endovenous ablation therapy of her right great saphenous vein (GSV) using CAC at an outside center four months before her presentation. The procedure was reportedly aborted after the first injection due to the extension of glue into the right common femoral vein (CFV). The operative report did not indicate the type of glue but mentioned that the catheter position was 8 cm away from the right saphenofemoral junction (SFJ). She has been on therapeutic anticoagulation, initially enoxaparin 60 mg twice daily subcutaneously, then rivaroxaban 20 mg daily since her procedure. A serial sonographic evaluation and a prolonged anticoagulation course were advised. She sought multiple second opinions and received the same advice.

**Figure 1:**
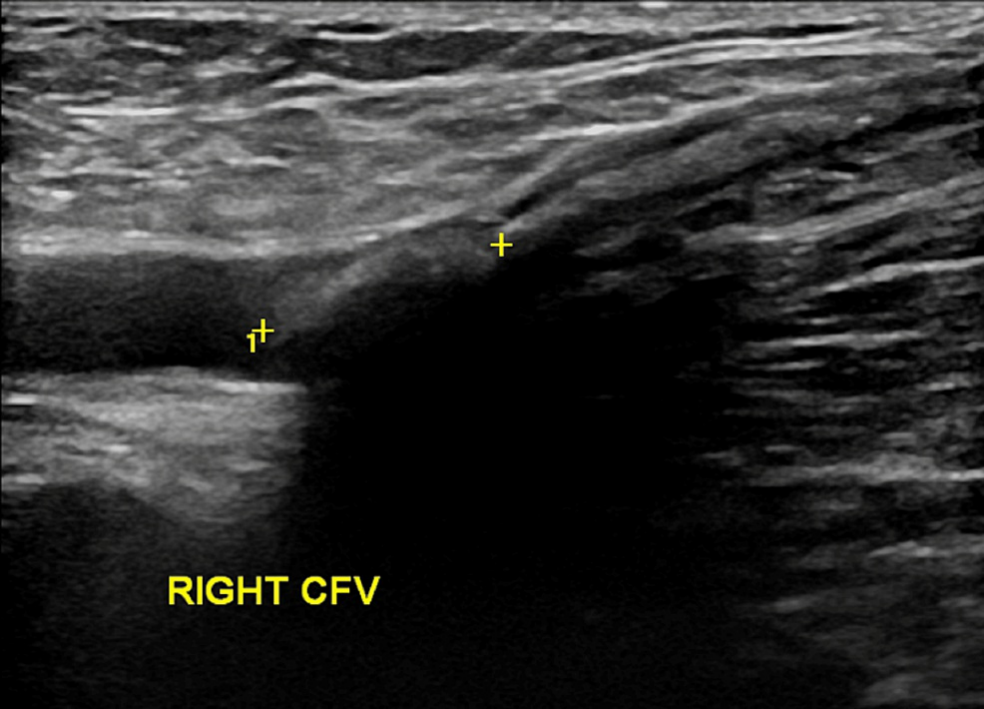
A preoperative Duplex scan showing glue extension into the right CFV CFV, common femoral vein

The patient presented with concerns about multiple bruises, episodes of epistaxis, and limited daily exercise and other activities due to anxiety and fear of bleeding. Her Duplex study showed glue extension into the right CFV, occupying over 50% of the transverse diameter of the lumen for a length of 13 mm (Figure 1), constituting a class III EGIT. Baseline postoperative images were not available for comparison. The distal segment of her right GSV was incompetent up to her knee. She wished to stop anticoagulation due to the physical and psychological burdens she was experiencing. High ligation of the GSV, as well as extraction of glue and potential repair or reconstruction of the CFV, was offered after counseling was provided to the patient. Operative risks, including deep venous thrombosis (DVT), bleeding, and potential injury to adjacent structures, were discussed, along with the risks of continuing anticoagulation therapy.

**Figure 2:**
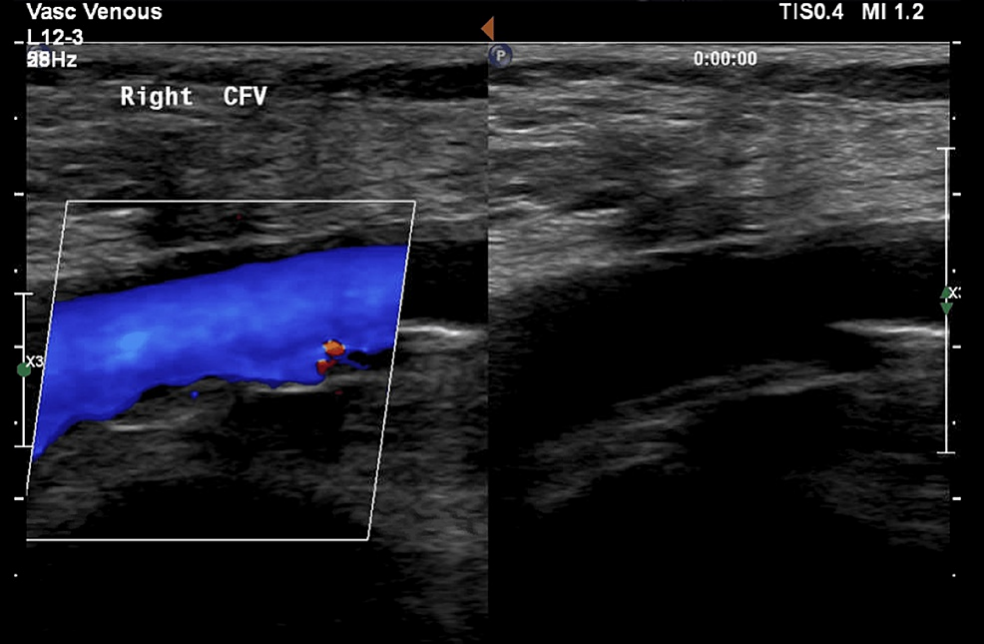
A patent right CFV 30 months after surgery CFV, common femoral vein

The patient underwent exploration and control of the right CFV. A longitudinal venotomy across the SFJ was extended superiorly along the right CFV after systemic heparinization. The removal of the foreign body (CAC polymers) was completed. The anterior wall of the right CFV had extensive fibrosis, which caused significant stenosis. This area was excised, and the defect was repaired using a bovine pericardial patch. High ligation of the right GSV was then completed. The intraoperative Duplex showed normal flow along the CFV after repair. The patient tolerated the procedure well and was discharged the following day on aspirin 81 mg once daily for three months. She returned for follow-up after one month and had a healed wound without signs of DVT. A Duplex scan showed a widely patent right CFV. Follow-up Duplex showed a patent right CFV at three, six, 18, and 30 months (Figure 2).

## Discussion

Endovenous saphenous ablation with CAC glue is a widely used technique with a similar closure rate to that of thermal modalities [3,4]. The classification of EGIT has been proposed by Cho et al. and is similar to the endovenous heat-inducted thrombosis classification of thermal ablation techniques [2]. EGIT has been reported in 5.8-21% of patients since the first human use of CAC in case reports and serial studies, but all reported patients had Duplex surveillance and resolution without any further intervention [1,4-6]. However, Parsi et al. recently published reports describing adverse events in CAC glue ablation cases that were submitted to the respected authorities in Australia, the United Kingdom, and the United States, which showed 211 thromboembolic events and seven strokes [7]. The young patient reported here had severe anxiety and significant limitations to her physical activities due to the use of anticoagulants for her class III (occlusion of over 50% of CFV lumen) EGIT. After multiple counseling visits, she opted for surgical treatment in order to stop her ongoing anticoagulation therapy. The high ligation and stripping of saphenous veins procedure has been the standard treatment for chronic venous insufficiency for many years with high success and low complication rates and remains a valuable option for selected patients [8].

## Conclusions

EGIT is a known complication of saphenous vein ablation using CAC, which requires anticoagulation therapy in severe forms. Surgical high ligation is a low-risk treatment option for patients who cannot tolerate anticoagulation treatment.
